# Comparative Microarray Analysis of Proliferating and Differentiating Murine ENS Progenitor Cells

**DOI:** 10.1155/2016/9695827

**Published:** 2015-11-30

**Authors:** Peter Helmut Neckel, Roland Mohr, Ying Zhang, Bernhard Hirt, Lothar Just

**Affiliations:** Institute of Clinical Anatomy and Cell Analysis, University of Tübingen, Österbergstrasse 3, 72074 Tübingen, Germany

## Abstract

Postnatal neural progenitor cells of the enteric nervous system are a potential source for future cell replacement therapies of developmental dysplasia like Hirschsprung's disease. However, little is known about the molecular mechanisms driving the homeostasis and differentiation of this cell pool. In this work, we conducted Affymetrix GeneChip experiments to identify differences in gene regulation between proliferation and early differentiation of enteric neural progenitors from neonatal mice. We detected a total of 1333 regulated genes that were linked to different groups of cellular mechanisms involved in cell cycle, apoptosis, neural proliferation, and differentiation. As expected, we found an augmented inhibition in the gene expression of cell cycle progression as well as an enhanced mRNA expression of neuronal and glial differentiation markers. We further found a marked inactivation of the canonical Wnt pathway after the induction of cellular differentiation. Taken together, these data demonstrate the various molecular mechanisms taking place during the proliferation and early differentiation of enteric neural progenitor cells.

## 1. Introduction

The enteric nervous system (ENS) is a largely autonomous and highly complex neuronal network found in the gastrointestinal tract. Its two major plexuses are integrated into the layered anatomy of the gut wall and, together with central modulating influences, exert control over gastrointestinal motility, secretion, ion-homeostasis, and immunological mechanisms [1]. In order to achieve this variety of functions, the ENS is composed of a multitude of different neuronal and glial cell types and closely interacts with smooth muscle cells and myogenic pacemaker cells called interstitial cells of Cajal. Furthermore, a population of neural stem or progenitor cells in the ENS has been identified in rodents [2, 3] and humans that retain their proliferative capacity throughout adult life even into old age [4, 5]. It is therefore not surprising that the correct functioning of the ENS as well as the regulation on enteric neural progenitor cells is subjected to the influence of a myriad of transmitters, neurotrophic and growth factors, signalling molecules, and extracellular matrix components, which are not exclusively expressed by neural cell types [6]. Likewise, the control of the development of the ENS is equally complex and mutations in its genetic program can lead to fatal dysplasia like Hirschsprung's disease (HCSR) [7, 8].

HSCR is hallmarked by an aganglionic distal bowel leading to life-threatening disturbances in intestinal motility. Today's therapeutic gold standard, the surgical resection of the affected gut segments, is nevertheless associated with problematic long-term outcomes with regard to continence [9]. In order to improve the therapeutic success, the use of autologous enteric neural stem cells was proposed [10]. This concept relies on the* in vitro* expansion of enteric neural stem cells derived from small biopsy materials. However, we are just beginning to understand the molecular mechanisms that underlie neural stem cell biology and how this knowledge can be used for optimizing* in vitro* culture conditions [11, 12].

Genome-wide gene-expression analyses are a useful tool to examine the genetic programs and cellular interactions and have been widely used to identify potential markers or signalling mechanisms especially in CNS neurospheres or cancer tissues. Further, gene-expression assays have also helped to unravel genetic prepositions associated with HSCR [13, 14], though little effort has so far been put into characterizing the genetic profile of enteric neural stem cells* in vitro* [15].

Here, we used an Affymetrix microarray analysis to evaluate the genetic expression profile of proliferating murine enteric neural stem cells and its changes during the early differentiation* in vitro*.

## 2. Materials and Methods

### 2.1. Cell Culturing

Cell culturing was conducted as described previously [15]. The handling of animals was in accordance to the institutional guidelines of the University of Tuebingen, which conform to the international guidelines.

**Figure 1:**
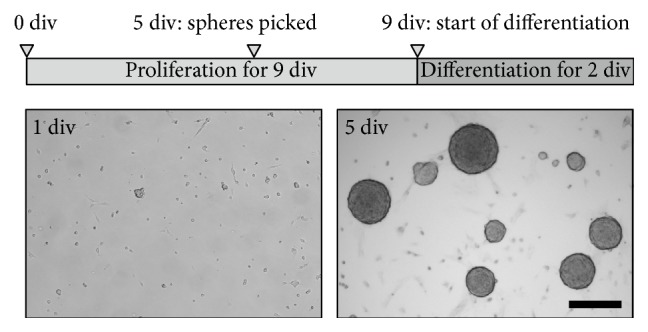
Time schedule of enterosphere culture. The timeline illustrates the schedule of* in vitro* culture. Cells were isolated at 0 div (days* in vitro*) and proliferated for 5 days. Spheres were then picked and again proliferated for 4 days. At 9 div, enterospheres were picked, washed, transferred to differentiation medium, and incubated for 2 days before gene expression analyses were carried out. The micrographs show proliferating enterospheres after 1 and 5 div. Scale bar: 200 *μ*m.

Neonatal (P0) C57BL/6 mice without regard to sex were decapitated and the whole gut was removed. After removal of adherent mesentery the longitudinal and circular muscle layers containing myenteric plexus could be stripped as a whole from the small intestine. Tissue was chopped and incubated in collagenase type XI (750 U/mL; Sigma-Aldrich, Taufkirchen, Germany) and dispase II (250 *μ*g/mL; Roche Diagnostics, Mannheim, Germany) dissolved in Hanks' balanced salt solution with Ca^2+^/Mg^2+^ (HBSS; PAA, Pasching, Austria) for 30 min at 37°C. During enzymatic dissociation the tissue was carefully triturated every 10 min with a fire polished 1 mL pipette tip. Prior to the first trituration step, cell suspension was treated with 0.05% (w/v) DNAse I (Sigma-Aldrich). After 30 min, tissue dissociation was stopped by adding fetal calf serum (FCS; PAA) to a final concentration of 10% (v/v) to the medium. Undigested larger tissue pieces were removed with a 40 *μ*m cell strainer (BD Biosciences, Franklin Lakes, NJ, USA). Residual enzymes were removed during two washing steps in HBSS at 200 g. After dissociation, cells were resuspended in proliferation culture medium (Dulbecco's modified Eagle's medium with Ham's F12 medium (DMEM/F12; 1 : 1; PAA)) containing N2 supplement (1 : 100; Invitrogen, Darmstadt, Germany), penicillin (100 U/mL; PAA), streptomycin (100 *μ*g/mL; PAA), L-glutamine (2 mM; PAA), epidermal growth factor (EGF; 20 ng/mL; Sigma-Aldrich), and fibroblast growth factor (FGF; 20 ng/mL; Sigma-Aldrich). Cells were seeded into 6-well plates (BD Biosciences) in a concentration of 2.5 × 10^4^ cells/cm^2^. Only once before seeding, the medium was supplemented with B27 (1 : 50; Invitrogen). EGF and FGF were added daily and culture medium was exchanged every 3 days. All cultivation steps were conducted in a humidified incubator at 37°C and 5% CO_2_. An overview of the following cell culture protocol is shown in Figure 1. During proliferation phase of the culture, cells formed spheroid-like bodies termed enterospheres. After 5 days of proliferation, free-floating enterospheres were picked and transferred to petri dishes (Ø 60 mm; Greiner Bio One, Frickenhausen, Germany) in 5 mL fresh proliferation medium and proliferation was continued for further 4 days.

Single free-floating enterospheres (50 enterospheres/dish) were picked again, washed 3 times in Tris buffer, and transferred into new petri dishes containing either proliferation medium or differentiation medium. Differentiation medium consists of DMEM/F12 containing N2 supplement (1 : 100), penicillin (100 U/mL), streptomycin (100 *μ*g/mL), L-glutamine (2 mM), and ascorbic acid-2-phosphate (200 *μ*M; Sigma-Aldrich).

Enterospheres were proliferated or differentiated for 2 more days, thereby forming the two experimental groups “proliferation” and “differentiation.” The difference in expression between those two groups (differentiation versus proliferation) was successively compared by microarray analysis as described below.

### 2.2. Affymetrix Microarray Analysis

Affymetrix microarray analysis was conducted similar to previously published data in three independent experiments, each with cell cultures prepared from 2 pups from the same litter [15]. In each experiment, free-floating enterospheres were picked as described above in order to diminish the fraction of adhesive fibroblasts and smooth muscle cells.

Total RNA of enterospheres of both groups was extracted using the RNeasy Micro Kit (Qiagen). RNA quality was evaluated on Agilent 2100 Bioanalyzer with RNA integrity numbers (RIN) of the samples in this study being in the range from 8 to 10. RIN numbers higher than 8 are considered optimal for downstream application [16].

Double-stranded cDNA was synthesized from 100 ng of total RNA, subsequently linearly amplified, and biotinylated using the GeneChip WT cDNA Synthesis and Amplification Kit (Affymetrix, Santa Clara, CA, USA) according to the manufacturer's instructions. 15 *μ*g of labeled and fragmented cDNA was hybridized to GeneChip Mouse Gene 1.0 ST arrays (Affymetrix). After hybridization, the arrays were stained and washed in a Fluidics Station 450 (Affymetrix) with the recommended washing procedure. Biotinylated cDNA bound to target molecules was detected with streptavidin-coupled phycoerythrin, biotinylated anti-streptavidin IgG antibodies and again streptavidin-coupled phycoerythrin according to the protocol. Arrays were scanned using the GCS3000 GeneChip Scanner (Affymetrix) and AGCC 3.0 software. Scanned images were subjected to visual inspection to check for hybridization artifacts and proper grid alignment and analyzed with Expression Console 1.0 (Affymetrix) to generate report files for quality control.

Normalization of raw data was performed by the Partek Software 6.6, applying an RMA (Robust Multichip Average) algorithm. Significance was calculated using a *t*-test without multiple testing correction (Partek), selecting all transcripts with a minimum change in expression level of 1.5-fold together with a *p* value less than 0.05.

## 3. Results

In this study, we investigated the changes of the genetic expression profile that occur during the transition from proliferating to differentiating enteric neural progenitor cells* in vitro*. Therefore, we generated enterospheres by 9 day* in vitro* cultures, which then could be picked and either proliferated or differentiated for two more days (Figure 1). mRNA was subsequently extracted and gene expression of these two groups was analysed by Affymetrix microarray analysis.

Analysis of mRNA expression was performed on a GeneChip Mouse Gene 1.0 ST array that determines the expression profile of 28.853 genes. Each gene was interrogated by a median of 27 probes that are spread along the full gene.

In total, the gene chip detected 1454 transcripts to be at least 1.5-fold differentially expressed between proliferating and differentiating enterospheres. 1333 of these transcripts code for already identified proteins. 541 genes were found to be upregulated and 792 genes were found to be downregulated in comparison to proliferating enterospheres (see Supplementary Table 1 of the Supplementary Material available online at http://dx.doi.org/10.1155/2016/9695827).

**Figure 2:**
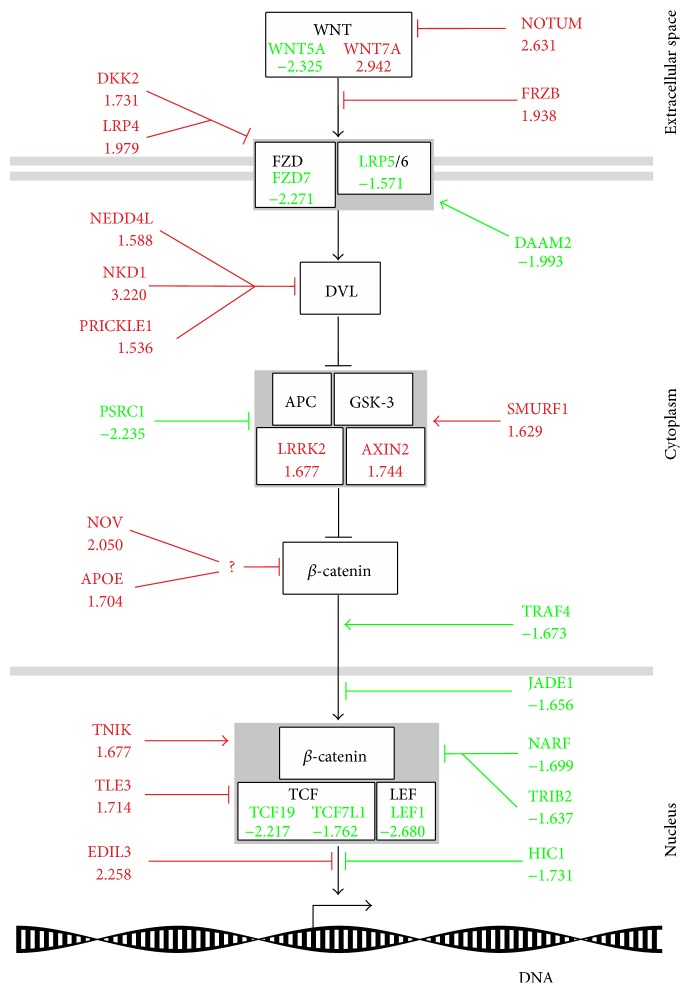
Detected regulatory influences on the canonical Wnt pathway. Scheme of the canonical Wnt pathway. Pointy arrowheads indicate an activating, blunt arrowheads, an inhibitory influence. The fold-change in expression of genes is written under the respective gene acronyms and colours indicate a general upregulation (red) or downregulation (green). For detailed explanation of the signalling cascade and regulated genes, see text.

We used the ingenuity pathway analysis software (IPA) and data mining with the science literature search engine http://www.ncbi.nlm.nih.gov/pubmed/ to divide the genes into different groups according to their function during cellular development. The largest functional group contained 171 genes related to cell cycle and apoptosis (Table 1, Supplementary Table 2). Here, we identified especially different cyclin proteins and cell division cycle proteins that were mainly downregulated. Further, we found several genes that are linked to neural development as well as genes regulating neural stem cell proliferation and differentiation. Furthermore, we also detected neuronal and glial differentiation markers and numerous genes involved in synapse formation (Table 2). It is noteworthy that we also identified a group of genes that are known to be involved in the differentiation of smooth muscle cells (Table 3) as well as in extracellular matrix components (Table 4). Additionally, we found regulated genes related to canonical Wnt signalling indicating a deactivation of this pathway during ENS progenitor cell differentiation (Figure 2, Table 5).

## 4. Discussion

The proliferation and differentiation of enteric neural progenitor cells during embryonic and postnatal development are controlled by a complex interplay of various intrinsic and extrinsic factors. Their exact timing is crucial for proper migration and proliferation of neural crest cells and for their differentiation into the various neural cell types that compose the complex neural structures of the ENS. Although research in recent years extended our understanding of ENS development and its pathologies [13], there are still many genes and processes unknown. Particularly, factors regulating neural progenitor proliferation and differentiation in the developing and postnatal gut as well as cellular and molecular interaction systems remain largely elusive. Here, we used* in vitro* cultures of enteric neural progenitor cells derived from murine tunica muscularis to scan for molecular programs and signalling pathways acting on cell proliferation and early differentiation.

Our experiment aimed to elucidate gene regulations in enterospheres that occur while ENS progenitor cells leave their proliferative state and begin to differentiate into more defined and specific cell types. The results of the Affymetrix gene expression analysis showed the up- and downregulation of overall 1333 known genes that code for already identified proteins. 171 of these genes could be linked to cell proliferation (Table 1, Supplementary Table 1). Amongst them we detected genes coding for proteins related to the kinetochore complex (like NSL1 [17], NUF2 [18], SKA1-3 [19], and ZWILCH [20]), cyclin proteins [21], cyclin-dependent kinases (CDK) [22], and several types of centromere proteins. The regulation of 145 of these genes strongly indicates a slowdown of cell cycle progression as it was intended by the experimental deprivation of growth factor supplementation by the end of the proliferation phase (see Section 3). Interestingly, betacellulin (BTC) was upregulated nearly 6-fold although it was reported to promote cellular proliferation in the neural stem cell niche [23]. Nonetheless, the vast majority of genes including all regulated cyclins, cell division cycle proteins, and kinetochore proteins were found to be downregulated.

We also checked the regulated genes for apoptosis markers to see whether the stop in proliferation was related to cell death (Supplementary Table 2). Since only 3 of 12 apoptotic genes were regulated in the direction that indicates apoptosis, it is unlikely that apoptosis played a leading role in the interruption of proliferation. Still, the effect and regulation of apoptosis during enteric sphere cultures are an important cornerstone of understanding enteric neural progenitors in culture and* in vivo* and require further investigation. Together, on a broad basis, this dataset provides strong evidence that this cell culture design is applicable to decreasing the proliferative rate of enteric neural progenitor cells without inducing cell death or apoptosis in an appreciable quantity.

To further evaluate the proliferative conditions of cell types present in enterospheres, we focused on different cell specific markers of neural progenitors as well as neurons, glial, or smooth muscle cells. We consider this complex cellular composition of the enterospheres an advantage compared to more purified neural crest derived neurospheres as we are able to capture complex interactions and secretion mechanisms between cell types that might also play an important role* in vivo*. Interestingly, we found 8 genes involved in adult central or embryonic neural stem cells homeostasis (Table 2). The majority of genes like EPHA2 [24] are regulated in a way that suggests that neural stem cells exit the proliferative cell cycle to enter differentiation programs. This idea was supported by the upregulation of numerous genes that drive neuronal and glial differentiation like NEUROD4 [25] or OLIG1 [26]. In this context, we identified several upregulated genes involved in proper myelination. As enteric and central glia cells are known to temporally express myelin-related proteins during development, it is conceivable that this regulation is part of the early glial differentiation program [27]. Moreover, also typical markers of differentiated neurons (class III beta-tubulin, CALB2 [28]) and enteric glia (GFAP [29]) were found to be upregulated. Intriguingly, S100B, a common glia cell marker, was downregulated contrasting the rest of our data. Again, this might be due to the complex differentiation program of enteric glia, in which S100B plays a role at later stages.

Furthermore, the establishment of neuronal cell communication was strongly regulated. Here, we found an increased expression of genes related to synaptogenesis (LRRTM2 and 3 [30], neurotrimin [31]) and to SNARE or vesicle protein function (STXBP3, SV2C [32], and SYT6 [33]). We also identified a number of genes involved in transmitter metabolism (COMT, DDC) as well as neurotransmitter receptor like 5-HT, glutamate, and adrenergic receptors. However, the regulation of those genes was highly variable shedding light on the intricacy of synapse formation in the developing enteric nervous system. This complexity is carried on by genes related to axon sprouting and guidance like semaphorins [34] or RGMa [35].

Additionally, we found that regulated genes directly involved in the differentiation of muscle cells and/or enteric pacemaker cells called interstitial cells of Cajal (Table 3). Particularly interesting is the upregulation of a number of genes known to drive smooth muscle differentiation like ARID5B [36], FOSL2 [36] and genes that are expressed in differentiated smooth muscle cells in the intestine like AFAP1 [37], ENPP2 [38], and CNN1 [39] as well as various myosin and actin isoforms. These data confirm the fact that cultured spheroids are composed of different cell types present in the intestinal tunica muscularis and further indicate that deprivation of growth factors induces differentiation of smooth muscle cells resembling molecular processes in the developing gut. In fact, we among others were previously able to confirm the presence of smooth muscle cells derived from enterosphere culture by BrdU-immunolabeling costudies [4]. However, it is noteworthy that a few genes related to muscular differentiation (endoglin [40], smoothelin [41], NUP210 [42], caldesmon 1 [43], and ACTN1 [44]) were downregulated contrasting the expression pattern observed in the majority of regulated genes. This hints to complex regulatory mechanisms controlling the myogenic differentiation program in which these genes are not required at all or in a different temporal sequence not mapped by our experimental design. It is further remarkable that five markers expressed in interstitial cells of Cajal (ICC) including KIT [45] were downregulated.

Moreover, the regulation of 43 extracellular matrix proteins like collagens, integrins, proteoglycans, and matrix metallopeptidases points to a reconstruction of extracellular environment that has been discussed to influence neural stem cell behaviour [46] (Table 4). Taken together, these results illustrate the ongoing genetic programs during early differentiation of enterospheres.

Within the dataset, it was of special interest to find particularly many regulated genes related to the canonical Wnt pathway (Table 5). The involvement of canonical Wnt signalling has frequently been shown in the regulation of various stem cell niches, like intestinal epithelium or CNS derived neural stem cells. However, these studies exhibited different and partly contradicting outcomes, which strongly hint to the variable functions of canonical Wnt signals in different tissues during embryonic and postnatal development. In previous work, we found regulation of several Wnt-related genes in the context of thyroid hormone dependent differentiation of enteric neural progenitor cells indicating a potential role of the canonical Wnt pathway activation during the proliferation of this progenitor cell pool [15]. Canonical Wnt signalling has frequently been reviewed in the literature—just recently by Ring et al. [47]. In brief, secreted Wnt proteins bind to frizzled receptors (FZD) complexed with low density lipoprotein receptor-related protein 5/6 (LRP5/6) coreceptors. Thereafter, the scaffolding protein disheveled (DVL) is recruited to FZD and inhibits the *β*-catenin destruction complex (AXIN2, APC, and GSK-3*β*). Therefore, *β*-catenin accumulates in the cytoplasm and translocates to the nucleus where it binds to TCF/LEF transcription factors to initiate Wnt target gene expression. Interestingly, our current data strongly indicate that the canonical Wnt pathway is switched off during the first two days of enteric progenitor differentiation on several levels of the signalling cascade (Figure 2). On the one hand we identified a downregulation of activating parts of the signalling cascade itself like the receptor proteins FZD7 and LRP5 or the transcription factors TCF19, TCF7L1, and LEF1. On the other hand, inactivating elements of the pathway like parts of the *β*-catenin destruction complex AXIN2 and LRRK2 [48] were upregulated. We also found numerous modulators of the signalling cascade. It is of interest that the majority of those genes are reported to inhibit the signalling process extracellularly or on receptor level (Notum [49], FRZB [50], DKK2 [51], and LRP4 [52]), in the cytoplasm (NEDD4L [53], NKD1 [54], PRICKLE1 [55], NOV [56], and APOE [57]), or in the nucleus (TLE3 [58], EDIL3 [59]). Furthermore, we identified target genes of the canonical Wnt pathway that were either upregulated (e.g., AXIN2 that exerts a negative feedback on the pathway) or downregulated like the cell cycle progression genes CCND1 and SPRY4 [60]. We also found a lower expression of SPRY2 [61], a Wnt target gene and known inhibitor of GDNF signalling [62], in the differentiation group. Together with a strong upregulation of GDNF itself by 4.325-fold, this might drive enteric progenitor cells into neural differentiation [12].

Taken together, it is conceivable that canonical Wnt signalling plays a role in the maintenance of the enteric progenitor pool during proliferation and is switched off at the beginning of differentiation conditions. Indeed, our previous gene expression analyses [15] as well as recently published cell culture experiments [63] and yet unpublished* in vitro* analyses strongly support this hypothesis.

## 5. Conclusion

This study focused on the changes in gene expression of enteric neural progenitor cells occurring within the first two days of transition from a proliferative state to differentiation* in vitro*. Using microarray analysis, we found a marked inhibition of cell cycle progression in general as well as strong evidence for neural stem cells differentiation into enteric neurons and glia cells. These findings were substantiated by the upregulation of genes related to synapse formation and neural connectivity. Most interesting, we found that this transition from enteric neural progenitor proliferation to differentiation was accompanied by a considerable inactivation of the canonical Wnt signalling pathway. This, together with previous work, strongly indicates that canonical Wnt activation is one of the driving mechanisms of enteric neural progenitor proliferation and thus might play a role in the homeostasis of this cell pool* in vivo* and* in vitro*.

## Supplementary Material

The supplementary material contains tables with additional information about the gene expression analysis.

## Figures and Tables

**Table 1:** Selected genes related to cell cycle.

Gene	Encoded protein	Fold change	Cell cycle
AURKA	Aurora kinase A	−2.712	**STOP**
AURKB	Aurora kinase B	−4.146	**STOP**
CCNA2	Cyclin A2	−4.652	**STOP**
CCNB1	Cyclin B1	−5.752 −5.820 −5.857	**STOP**
CCNB2	Cyclin B2	−3.392	**STOP**
CCND1	Cyclin D1	−2.476	**STOP**
CCND3	Cyclin D3	−1.539	**STOP**
CCNE1	Cyclin E1	−1.777	**STOP**
CCNE2	Cyclin E2	−2.847	**STOP**
CCNF	Cyclin F	−3.211	**STOP**
CDC6	Cell division cycle 6	−1.936	**STOP**
CDC20	Cell division cycle 20	−3.113	**STOP**
CDC25B	Cell division cycle 25B	−1.636	**STOP**
CDC25C	Cell division cycle 25C	−2.414	**STOP**
CDC45	Cell division cycle 45	−1.769	**STOP**
CDCA2	Cell division cycle associated 2	−3.461	**STOP**
CDCA3	Cell division cycle associated 3	−3.003	**STOP**
CDCA5	Cell division cycle associated 5	−3.053	**STOP**
CDCA7L	Cell division cycle associated 7-like	−4.123	**STOP**
CDCA8	Cell division cycle associated 8	−3.467	**STOP**
CDK1	Cyclin-dependent kinase 1	−3.227	**STOP**
CDK15	Cyclin-dependent kinase 15	1.618	*GO *
CDK19	Cyclin-dependent kinase 19	1.619	*GO *
CDK5R1	Cyclin-dependent kinase 5, regulatory subunit 1 (p35)	1.597	—
CENPA	Centromere protein A	−1.895	**STOP**
CENPE	Centromere protein E, 312 kDa	−4.140	**STOP**
CENPF	Centromere protein F, 350/400 kDa	−3.927	**STOP**
CENPI	Centromere protein I	−2.899	**STOP**
CENPK	Centromere protein K	−2.813	**STOP**
CENPL	Centromere protein L	−1.864	**STOP**
CENPM	Centromere protein M	−3.407	**STOP**
CENPN	Centromere protein N	−2.465	**STOP**
CENPU	Centromere protein U	−1.624	**STOP**
SKA1	Spindle and kinetochore associated complex subunit 1	−1.532	**STOP**
SKA2	Spindle and kinetochore associated complex subunit 2	−1.582	**STOP**
SKA3	Spindle and kinetochore associated complex subunit 3	−3.490	**STOP**
SKP2	S-phase kinase-associated protein 2, E3 ubiquitin protein ligase	−1.845	**STOP**
SPC25	SPC25, NDC80 kinetochore complex component	−4.148	**STOP**

**Table 2:** Neural differentiation/development.

Gene	Encoded protein	Fold change
*Neural stem cells *		
ABCG2	ATP-binding cassette, subfamily G (WHITE), member 2 (junior blood group)	−1.526
ASPM	asp (abnormal spindle) homolog, microcephaly associated (*Drosophila*)	−4.911
CDT1	Chromatin licensing and DNA replication factor 1	−1.528
EGFL7	EGF-like-domain, multiple 7	3.132
EPHA2	EPH receptor A2	−1.529
ETV4	ets variant 4	−1.934
ETV5	ets variant 5	−2.844
		−2.651
FABP7	Fatty acid binding protein 7, brain	−2.095

*Neural differentiation *		
ATOH8	Atonal homolog 8 (*Drosophila*)	1.932
AXL	AXL receptor tyrosine kinase	2.015
CRIM1	Cysteine-rich transmembrane BMP regulator 1 (chordin-like)	1.999
CRLF1	Cytokine receptor-like factor 1	2.382
DAB1	Dab, reelin signal transducer, homolog 1 (*Drosophila*)	−2.297
ELK3	ELK3, ETS-domain protein (SRF accessory protein 2)	−1.613
ESCO2	Establishment of sister chromatid cohesion N-acetyltransferase 2	−4.767
GAP43	Growth associated protein 43	1.613
GLDN	Gliomedin	5.809
HMOX1	Heme oxygenase (decycling) 1	1.884
KLF9	Kruppel-like factor 9	1.592
Lmo3	LIM domain only 3	1.542
MAP6	Microtubule-associated protein 6	1.874
MYRF	Myelin regulatory factor	2.527
NEUROD4	Neuronal differentiation 4	2.036
OLIG1	Oligodendrocyte transcription factor 1	2.660
Pvr	Poliovirus receptor	1.768
RGS4	Regulator of G-protein signaling 4	1.955
S1PR1	Sphingosine-1-phosphate receptor 1	5.073
SOCS2	Suppressor of cytokine signaling 2	2.052
		2.335
WIPF1	WAS/WASL interacting protein family, member 1	1.587

*Neural differentiation markers *		
CALB2	Calbindin 2	1.616
CNP	2′,3′-Cyclic nucleotide 3′-phosphodiesterase	1.732
GFAP	Glial fibrillary acidic protein	2.239
MBP	Myelin basic protein	1.768
Mturn	Maturin, neural progenitor differentiation regulator homolog (*Xenopus*)	1.853
OMG	Oligodendrocyte myelin glycoprotein	−1.822
OPALIN	Oligodendrocytic myelin paranodal and inner loop protein	39.246
PLP1	Proteolipid protein 1	1.630
S100B	S100 calcium binding protein B	−1.675
TUBB2A	Tubulin, beta 2A class IIa	1.608
TUBB2B	Tubulin, beta 2B class IIb	1.535
TUBB3	Tubulin, beta 3 class III	1.976

*Synapse and neurotransmitters *		
ABAT	4-Aminobutyrate aminotransferase	−1.512
ADRA1D	Adrenoceptor alpha 1D	1.803
ADRA2A	Adrenoceptor alpha 2A	2.900
ADRA2B	Adrenoceptor alpha 2B	−2.093
CHRM2	Cholinergic receptor, muscarinic 2	1.635
CHRM3	Cholinergic receptor, muscarinic 3	−1.715
CHRNA7	Cholinergic receptor, nicotinic, alpha 7 (neuronal)	1.772
COMT	Catechol-O-methyltransferase	1.515
DDC	DOPA decarboxylase (aromatic L-amino acid decarboxylase)	1.711
DNM3	Dynamin 3	2.643
EPHA5	EPH receptor A5	2.076
GRIA3	Glutamate receptor, ionotropic, AMPA 3	−1.528
GRIA4	Glutamate receptor, ionotropic, AMPA 4	−1.997
GRIK2	Glutamate receptor, ionotropic, kainate 2	−1.565
GRM5	Glutamate receptor, metabotropic 5	−1.600
HTR1B	5-Hydroxytryptamine (serotonin) receptor 1B, G-protein-coupled	−2.377
HTR2B	5-Hydroxytryptamine (serotonin) receptor 2B, G-protein-coupled	2.205
LRRTM2	Leucine-rich repeat transmembrane neuronal 2	3.665
LRRTM3	Leucine-rich repeat transmembrane neuronal 3	2.210
NTM	Neurotrimin	1.820
PENK	Proenkephalin	3.478
PRR7	Proline rich 7 (synaptic)	1.788
SLC10A4	Solute carrier family 10, member 4	1.824
		1.867
SLITRK2	SLIT and NTRK-like family, member 2	−2.414
SLITRK6	SLIT and NTRK-like family, member 6	1.672
STON2	Stonin 2	4.054
STXBP3	Syntaxin-binding protein 3	1.730
Stxbp3b	Syntaxin-binding protein 3B	1.637
SV2C	Synaptic vesicle glycoprotein 2C	1.929
SYT6	Synaptotagmin VI	2.571

*Neurite outgrowth *		
ATF3	Activating transcription factor 3	2.579
DOK4	Docking protein 4	4.937
FEZ2	Fasciculation and elongation protein zeta 2 (zygin II)	1.547
NAV2	Neuron navigator 2	1.647
NRCAM	Neuronal cell adhesion molecule	2.496
PLXNB3	Plexin B3	1.739
RGMA	Repulsive guidance molecule family member a	1.552
RNF165	Ring finger protein 165	−1.548
ROBO2	Roundabout, axon guidance receptor, homolog 2 (*Drosophila*)	−2.211
SEMA3B	Sema domain, immunoglobulin domain (Ig), short basic domain, secreted (semaphorin) 3B	3.692
SEMA3E	Sema domain, immunoglobulin domain (Ig), short basic domain, secreted (semaphorin) 3E	2.877
SEMA4F	Sema domain, immunoglobulin domain (Ig), transmembrane domain (TM) and short cytoplasmic domain, (semaphorin) 4F	4.891
SEMA6A	Sema domain, transmembrane domain (TM), and cytoplasmic domain (semaphorin) 6A	−1.707
SRGAP1	SLIT-ROBO Rho GTPase activating protein 1	1.524
UNC5B	unc-5 homolog B (*C. elegans*)	−1.927

*Growth factors *		
ARTN	Artemin	2.423
FGF2	Fibroblast growth factor 2 (basic)	2.264
FGF5	Fibroblast growth factor 5	7.704
GDF10	Growth differentiation factor 10	−2.361
GDF11	Growth differentiation factor 11	1.604
GDNF	Glial cell derived neurotrophic factor	4.325
GFRA3	GDNF family receptor alpha 3	1.707
MET	MET protooncogene, receptor tyrosine kinase	6.680
NGFR	Nerve growth factor receptor	1.728
NTRK3	Neurotrophic tyrosine kinase, receptor, type 3	−1.575
SNX16	Sorting nexin 16	1.641
SPHK1	Sphingosine kinase 1	1.704
SPRY1	Sprouty homolog 1, antagonist of FGF signaling (*Drosophila*)	−1.647

**Table 3:** Differentiation of smooth muscle cells/ICCs.

Gene	Encoded protein	Fold change
*Smooth muscle cells *		
ACTA2	Actin, alpha 2, smooth muscle, aorta	1.693
ACTG2	Actin, gamma 2, smooth muscle, enteric	2.336
ACTN1	Actinin, alpha 1	−1.724
AEBP1	AE binding protein 1	2.702
AFAP1	Actin filament associated protein 1	1.638
ARID5B	AT-rich interactive domain 5B (MRF1-like)	1.521
Cald1	Caldesmon 1	−1.535
CNN1	Calponin 1, basic, smooth muscle	1.652
ENG	Endoglin	−1.552
ENPP1	Ectonucleotide pyrophosphatase/phosphodiesterase 1	−1.522
ENPP2	Ectonucleotide pyrophosphatase/phosphodiesterase 2	2.959
ENTPD1	Ectonucleoside triphosphate diphosphohydrolase 1	1.636
FOSL2	FOS-like antigen 2	2.566
GAMT	Guanidinoacetate N-methyltransferase	1.725
MYO1E	Myosin IE	1.569
MYO5A	Myosin VA (heavy chain 12, myoxin)	1.680
MYO7B	Myosin VIIB	1.710
MYO18A	Myosin XVIIIA	1.994
MYPN	Myopalladin	1.570
NEB	Nebulin	1.569
Nebl	Nebulette	2.378
NUP210	Nucleoporin 210 kDa	−1.838
RBM24	RNA binding motif protein 24	1.548
SMTN	Smoothelin	−1.778
SSPN	Sarcospan	1.603
TAGLN	Transgelin	2.706

*ICC *		
GUCY1A3	Guanylate cyclase 1, soluble, alpha 3	−1.876
GUCY1B3	Guanylate cyclase 1, soluble, beta 3	−2.008
KIT	v-kit Hardy-Zuckerman 4 feline sarcoma viral oncogene homolog	−1.798
KITLG	KIT ligand	−1.541

**Table 4:** ECM.

Gene	Encoded protein	Fold change
CHSY3	Chondroitin sulfate synthase 3	−1.645
COL6A5	Collagen, type VI, alpha 5	1.527
COL12A1	Collagen, type XII, alpha 1	−1.973
COL14A1	Collagen, type XIV, alpha 1	6.135
COL16A1	Collagen, type XVI, alpha 1	1.666
COL18A1	Collagen, type XVIII, alpha 1	1.595
COL27A1	Collagen, type XXVII, alpha 1	1.522
COLGALT2	Collagen beta(1-O)galactosyltransferase 2	−1.564
CSPG4	Chondroitin sulfate proteoglycan 4	−2.952
CSPG5	Chondroitin sulfate proteoglycan 5 (neuroglycan C)	−1.585
CYR61	Cysteine-rich, angiogenic inducer, 61	1.748
ECM1	Extracellular matrix protein 1	2.580
HSPG2	Heparan sulfate proteoglycan 2	1.923
ITGA1	Integrin, alpha 1	−1.665
ITGA4	Integrin, alpha 4 (antigen CD49D, alpha 4 subunit of VLA-4 receptor)	−2.324
ITGA7	Integrin, alpha 7	4.203
ITGA8	Integrin, alpha 8	−2.262
ITGA11	Integrin, alpha 11	1.762
ITGB3	Integrin, beta 3 (platelet glycoprotein IIIa, antigen CD61)	−5.342
ITGB4	Integrin, beta 4	1.567
KRT80	Keratin 80	2.833
LAMA4	Laminin, alpha 4	−1.537
LAMA5	Laminin, alpha 5	1.684
LOX	Lysyl oxidase	3.250
LOXL4	Lysyl oxidase-like 4	2.427
		2.417
MATN2	Matrilin 2	2.570
MMP2	Matrix metallopeptidase 2 (gelatinase A, 72 kDa gelatinase, 72 kDa type IV collagenase)	1.668
MMP9	Matrix metallopeptidase 9 (gelatinase B, 92 kDa gelatinase, 92 kDa type IV collagenase)	−5.557
MMP15	Matrix metallopeptidase 15 (membrane-inserted)	−2.017
MMP16	Matrix metallopeptidase 16 (membrane-inserted)	−1.634
MMP17	Matrix metallopeptidase 17 (membrane-inserted)	1.612
MMP19	Matrix metallopeptidase 19	3.236
MMP28	Matrix metallopeptidase 28	1.956
NDST3	N-deacetylase/N-sulfotransferase (heparan glucosaminyl) 3	−5.557
P4HA1	Prolyl 4-hydroxylase, alpha polypeptide I	−1.958
PLOD3	Procollagen-lysine, 2-oxoglutarate 5-dioxygenase 3	2.250
UGDH	UDP-glucose 6-dehydrogenase	1.529

**Table 5:** Wnt.

Gene	Encoded protein	Fold change
*Wnt signaling cascade *		
FZD7	Frizzled class receptor 7	−2.271
LEF1	Lymphoid enhancer-binding factor 1	−2.680
LRP5	Low density lipoprotein receptor-related protein 5	−1.571
LRRK2	Leucine-rich repeat kinase 2	1.677
TCF19	Transcription factor 19	−2.217
F7L1	Transcription factor 7-like 1 (T-cell specific, HMG-box)	−1.762
WNT5A	Wingless-type MMTV integration site family, member 5A	−2.325
WNT7B	Wingless-type MMTV integration site family, member 7B	2.942

*Target gene *		
ARL4C	ADP-ribosylation factor-like 4C	2.179
AXIN2	Axin 2	1.744
CCND1	Cyclin D1	−2.476
CSRNP1	Cysteine-serine-rich nuclear protein 1	1.822
RACGAP1	Rac GTPase activating protein 1	−3.201
SPRY2	Sprouty homolog 2 (*Drosophila*)	−1.771
SPRY4	Sprouty homolog 4 (*Drosophila*)	−2.771
WISP1	WNT1 inducible signaling pathway protein 1	2.489

*Wnt antagonists/inhibitors *		
APOE	Apolipoprotein E	1.704
DKK2	Dickkopf WNT signaling pathway inhibitor 2	1.731
EDIL3	EGF-like repeats and discoidin I-like domains 3	2.258
FRZB	Frizzled-related protein	1.938
HIC1	Hypermethylated in cancer 1	−1.731
JADE1	Jade family PHD finger 1	−1.656
LRP4	Low density lipoprotein receptor-related protein 4	1.979
NARF	Nuclear prelamin A recognition factor	−1.699
NEDD4L	Neural precursor cell expressed, developmentally downregulated 4-like, E3 ubiquitin protein ligase	1.588
NKD1	Naked cuticle homolog 1 (*Drosophila*)	3.220
NOTUM	Notum pectinacetylesterase homolog (*Drosophila*)	2.631
NOV	Nephroblastoma overexpressed	2.050
PRICKLE1	Prickle homolog 1 (*Drosophila*)	1.536
TLE3	Transducin-like enhancer of split 3	1.714
TRIB2	Tribbles pseudokinase 2	−1.637

*Wnt activators *		
DAAM2	Dishevelled associated activator of morphogenesis 2	−1.993
PSRC1	Proline/serine-rich coiled-coil 1	−2.235
TNIK	TRAF2 and NCK interacting kinase	1.677
TRAF4	TNF receptor-associated factor 4	−1.673
