# Supplementary material for: Comparative Microarray Analysis of Proliferating and Differentiating Murine ENS Progenitor Cells
Source: Stem Cells Int. 2015 Nov 30;2016:9695827. doi: 10.1155/2016/9695827 (PMC4677255; doi:10.1155/2016/9695827)
Supplement: Supplementary file 1 — The supplementary material contains tables with additional information about the gene expression analysis. [file 9695827.f1.pdf]

# Supplementary Tables

## Supplementary Table 1: All regulated genes

### Up-regulated Genes

5730471H19Rik, 6720489N17Rik, AAAS, ABAT, ABCA6, ABCC3, ABCG2, ACTN1, ADAM12, ADAMTS9, ADORA1, ADRA2B, AFMID, AGMO, Airn, AJAP1, AK4, ALDH3B1, ALG6, ALPL, AMOT, ANGPT2, ANGPTL1, ANKRD29, ANLN, ANO1, ANO3, ARAP2, ARHGAP11A, ARHGAP19, ARHGAP6, ARHGEF39, ARHGEF6, ARSB, ASF1B, ASPH, ASPM, ASPN, ATAD2, ATAD5, ATP10B, ATP1A2, ATP2A3, AURKA, AURKB, BARD1, BCHE, BCL6B, BIRC5, BORA, BRCA1, BRCA2, BRINP1, BRIP1, Btbd19, BUB1, BUB1B, C14orf159, C14orf37, C14orf80, C15orf39, C17orf53, C17orf58, C1QTNF1, C1QTNF7, C3orf67, C4orf46, CA12, CA6, CA8, Cald1, Calr4, CAMK1G, CARD6, CASC5, CAV1, CBR3, CCDC3, CCNA2, CCNB1, CCNB2, CCND1, CCND3, CCNE1, CCNE2, CCNF, CD200, CD38, CD40, CD97, CDC20, CDC25B, CDC25C, CDC45, CDC6, CDCA2, CDCA3, CDCA5, CDCA7L, CDCA8, CDK1, CDKN2C, CDKN3, CDO1, CDRT4, CDT1, CEACAM1, CENPA, CENPE, CENPF, CENPI, CENPK, CENPL, CENPM, CENPN, CENPU, CEP55, CHAF1A, CHAF1B, CHCHD10, CHEK1, CHEK2, CHRM3, CHST7, CHSY3, CHTF18, CKAP2, CKAP2L, CKB, CKS1B, CKS2, CLDN15, CLEC14A, CLMP, CLSPN, CMC2, CMKLR1, CMTM8, COL12A1, COLGALT2, COTL1, COX7A1, CRYL1, CRYM, CRYZ, CSPG4, CSPG5, Ctla2a/Ctla2b, CTNNAL1, CXCL16, CYP51A1, CYSLTR1, D17H6S56E-5, DAAM2, DAB1, DAB2, DAGLA, DAPP1, DBF4, DBP, DCHS1, DCK, DDIAS, DDX11, DEPDC1, DEPDC1B, DHCR24, DHCR7, DHFR, DIAPH3, DLC1, DLGAP5, DNA2, DNPH1, DOCK10, DOCK11, DOCK6, DPEP2, DSCC1, DTL, DUSP4, DUSP6, E2F1, E2F2, E2F3, E2F7, E2F8, ECT2, EDA2R, EFCAB11, EGFL8, EGLN3, ELK3, ELMOD1, ELOVL2, EME1, ENG, ENO1, ENPEP, ENPP1, EPHA2, EPHX1, EPPIN, EPS8, ERCC6L, ERI2, ESCO2, ESPL1, ETV4, ETV5, EXO1, F3, FABP7, FAM107B, FAM110A, FAM111A, FAM122B, FAM174B, FAM212A, FAM212B, FAM64A, FAM83D, FANCA, FANCB, FANCD2, FANCI, FAT3, FBP1, FBXO48, FBXO5, FBXW7, FDFT1, FGD1, FHDC1, FIBIN, FIGNL1, FILIP1L, FJX1, FLI1, FLNB, FOXM1, FOXRED2, FZD7, G2E3, GALNT18, GAS5, GDF10, GEN1, GGT5, GLRX, GNAI1, GPBAR1, GPC3, GPC4, GPC6, GPD1, GPR116, GPR133, GPR182, GPRC5C, GRIA3, GRIA4, GRIK2, GRM5, GSG2, Gsta4, GSTK1, Gstt3, GTSE1, GUCY1A3, GUCY1B3, H2AFX, H2AFZ, HEATR5B, HELLS, HIC1, HIRIP3, Hist1h1a, Hist1h1b, HIST1H2AA, HIST1H2AD, HIST1H2AG, HIST1H2AJ, Hist1h2ap, HIST1H2BB, HIST1H2BF, HIST1H2BH, HIST1H2BI, HIST1H2BN, HIST1H3B, HIST1H3C, HIST1H3D, HIST1H3J, HIST1H4A, HIST1H4B, HIST1H4C, HIST1H4F, HIST1H4I, HIST1H4J, HIST1H4L, HIST2H2AA3/HIST2H2AA4, HIST2H2BF, HIST2H3A, HIST2H4A, HJURP, HMCN1, Hmga2, HMGB2, HMGCS1, HMMR, HOPX, HOXC6, HSD11B2, HTR1B, HVCN1, IDI1, IFI16, IFI30, Ifnz (includes others), IGF2R, IGFBP3, IL15, IL2RG, INCENP, INHBA, INPP4B, INSIG1, IQGAP3, ITGA1, ITGA4, ITGA8, ITGB3, ITPKB, ITPRIPL1, JADE1, JAM2, KANK1, KANK4, KBTBD6, KCNC1, KCNG4, KCNJ10, KCNJ2, KCNK5, KCNMB2, KCNMB4, Kcnn2, Kctd12b, KHDRBS3, KIAA0040, KIAA0101, KIAA1524, KIF11, KIF14, KIF15, KIF18A, KIF18B, KIF20A, KIF20B, KIF22, KIF23, KIF24, KIF2C, KIF4A, KIT, KITLG, KNSTRN, KNTC1, KPNA2, LAMA4, LBR, LDLRAD4, LEF1, LGALS3BP, LHFPL1, LHFPL3, LIG1, LIMCH1, LIN7A, LMNB1, LONRF3, LPAR4, LPHN2, LRP5, LRR1, LRRC55, LSS, LY96, LYN, MAD2L1, MAP2K6, MAPK12, MAST4, MASTL, MATN4, MCC, MCM10, MCM2, MCM3, MCM4, MCM5, MCM7, MCM8, MEF2C, MEGF10, MELK, MGAT4A, MIS18BP1, MKI67, MMD, MMP15, MMP16, MMP9, MMS22L, MNS1, MRO, MSH6, MSMO1, MTFR2, MVD, MXD3, MYBL1, MYBL2, NAALAD2, NABP1, NARF, NASP, NCAPD2, NCAPD3, NCAPG, NCAPG2, NCAPH, NDC80, NDST3, NEIL3, NEK2, NETO2, NKAIN4, NKX3-2, NOX4, NPPC, NQO2, NRP2, NSL1, NTRK3, NTSR1, NUF2, NUP210, NUSAP1, OIP5, OLFM2, OMG, OPCML, ORAI1, P2RY1, P4HA1, PACRG, PALD1, PARPBP, PASK, PBK, PCDH12, PDCD4, PDE3A, PDE3B, PDE7B, PDE8B, PDE9A, PDGFB, PDGFD, PDZD2, PEAK1, PELI1, PFKFB3, PFKL, PGM5, PHF19, PID1, PIDD1, PIK3CG, PLA2G4A, PLCB1, PLK1, PLK2, PLK4, PLLP, PLVAP, PNP, PNPLA3, POLA1, POLA2, POLD1, POLE, POLE2, POLH, Pou3f1, PPARGC1A, PRC1, PRICKLE2, PRIM1, PRKAR2B, PRKCQ, PRLR, PRR11, PSRC1, PTGFRN, PTGS1, PTH2R, PTPLB, PTPRM, PTPRO, PTPRZ1, PYGM, RAB18, RACGAP1, RAD51, RAD51AP1, RAD51B, RAD51C, RAD54B, RAD54L, RASA3, RASD2, RASGRP3, RBL1, RBPMS, RCSD1, REP15, RFC4, RFWD3, RND3, RNF165, ROBO2, RPL13, RRM1, RRM2, RTKN2, S100B, SAMD5, SASH1, SCRG1, SELM, SEMA6A, SEPP1, SEPT6, SERPINB9, SERTAD4, SGOL1, SGOL2, SGSH, SH2B3, SH3BGRL, SH3BGRL2, SH3BP1, SHC3, SHCBP1, SIPA1L1, SIX4, SKA1, SKA2, SKA3, SKP2, SLBP, SLC12A2, SLC16A3, SLC25A13, SLC25A35, SLC29A1, SLC35A1, SLC38A4, SLC43A3, SLFN13, SLITRK2, SMC2, SMC4, SMTN, SNCAIP, Snord116, SNX1, SNX22, SNX25, SORCS1, SOX5, SPAG5, SPC25, SPON2, SPRED2, SPRY1, SPRY2, SPRY4, ST3GAL6, ST5, STAG3, STAP2, STARD8, STEAP2, STIL, STK10, STOM, SYCE2, TACC3, TBC1D31, TCF19, TCF7L1, TENM4, TF, TFRC, TICRR, TIMP4, TK1, TLCD1, TLL1, TLL2, TLR3, TM7SF2, TMEM173, TMEM194A, TMEM204, TMEM229A, TMEM53, TMEM56, TMEM97, TMEM98, TMPO, TNFRSF10A, Tnfrsf22/Tnfrsf23, TNS1, TONSL, TOP2A, TOPBP1, TOR4A, TP53I11, TP53INP1, TPCN1, TPR, TPX2, TRAF4, TRHR, TRIB2, TRIL, TRIM59, TRIP13, TROAP, TRPC3, TSEN15, TSPAN18, TSPAN7, TSPAN9, TTK, TYMS, UBE2C, UBE2L6, UBE2T, UHRF1, UNC5B, USP1, VSNL1, WDHD1, WDR76, WEE1, WNT5A, XKR5, XRCC2, XYLT1, YPEL2, ZAK, ZCCHC16, ZCCHC3, ZDHHC15, ZDHHC2, Zfp760, ZFPM2, ZGRF1, ZMYND15, ZNF367, ZNF395, ZWILCH

**Down-regulated Genes**

9430069I07Rik, ABCA1, ABLIM2, ACKR3, Acot1, Acot6, ACSL3, ACTA2, ACTG2, ADAM11, ADAM9, ADAMTS17, ADAMTS19, ADAMTS3, ADAMTS7, ADAMTSL1, ADH1C, ADRA1D, ADRA2A, ADSSL1, AEBP1, AFAP1, AGPAT2, AGTR2, AHNAK, AHR, AHRR, AKAP6, ALDH1L2, ALX4, AMACR, ANXA2, ANXA4, ANXA7, APOE, AQP1, ARHGAP20, ARHGAP23, ARHGAP28, ARHGEF10, ARHGEF10L, ARHGEF19, ARID5B, ARL4C, ARRB1, ARSI, ARSJ, ARTN, AS3MT, ASPA, ASTN2, ASXL3, ATF3, ATOH8, AUH, AXIN2, AXL, BACE2, BCL3, Bhlhe41, BMF, BMPER, BTBD16, BTC, C1QL2, C1R, C4A/C4B, C4orf22, C4orf32, C8orf4, C9orf91, CACNG5, CADM4, CALB2, CAMK2A, CAPG, CASQ2, CCDC141, CD109, CD151, CD63, CDH13, CDK15, CDK19, CDK5R1, Cdsn, CEBPD, CELA1, CELF3, CELSR1, CFLAR, CHAC1, CHD3, CHMP4C, CHN2, CHODL, CHRM2, CHRNA7, CHST8, CLDN11, CLN8, CNKSR2, CNKSR3, CNN1, CNP, COL14A1, COL16A1, COL18A1, COL27A1, COL6A5, COMT, Cox8b, CPNE4, CPNE8, CRABP1, CRIM1, CRLF1, CRTAC1, CSRNP1, CTHRC1, CXCL14, CYP1B1, CYP2F1, CYP4B1, CYR61, DBNDD2, DCLRE1C, DDC, DDIT4L, DGAT2, DGKA, DHDH, DHRS3, DKK2, DNM3, Dnm3os, DOK4, DPEP1, DPT, DRAM1, Dst, DUSP1, DUSP10, DUSP14, DUSP8, EAPP, ECM1, EDIL3, EGFL7, EHBP1, EID1, ELFN2, ELMO1, EML1, ENPP2, ENPP4, ENPP6, ENTPD1, ENTPD2, EPDR1, EPHA5, EVA1C, FA2H, FADS3, FAM19A5, FAM213B, FAM78B, FAM89A, FBXL5, FBXO32, FEZ2, FGD3, FGF2, FGF5, FGL2, FIGF, FMO1, FOSL2, FRMD5, FRZB, FSTL3, FSTL4, FXYD3, FYB, GADD45A, GADD45B, GADD45G, GAL3ST2, GALNT16, GALNT9, GAMT, GAP43, GAS1, GATM, GBP2, GCNT2, GDF11, GDNF, GEM, GFAP, GFRA3, GGCT, GLDN, GLIS3, GPC1, GPNMB, GPR137C, GREB1, HFE, HMOX1, HPS1, HRSP12, HSPA1L, HSPA2, HSPG2, HTR2B, HTT, HYAL1, ID3, IER5, IGFBP2, IGSF10, IL16, IL17RC, IL34, IL3RA, IL4R, IL6R, INF2, INSC, IPO13, ITGA11, ITGA7, ITGB4, ITIH5, JDP2, JUN, KCNA1, KCNA4, KCNK13, KCNRG, KERA, KIAA0226, KIAA0930, KIAA1462, KIAA1614, KIRREL3, KLF9, KLHL30, KLHL34, KLK9, KRT80, LAMA5, LBP, LECT1, LGALS3, LGI3, LIF, LIMS2, Lmo3, LOX, LOXL4, LPCAT2, LPHN3, LPPR3, LRP4, LRRC8E, LRRK2, LRRTM2, LRRTM3, LRTM1, LUZP2, Ly6a (includes others), MAL, MANBA, MAP6, MAPKBP1, MATN2, MBP, MERTK, MET, MFAP3L, MFNG, MGLL, MICALL1, MMEL1, MMP17, MMP19, MMP2, MMP28, MREG, MRGPRF, MRPL33, MTR, Mturn, Muc1, MUSTN1, MVP, MYEOV2, MYL1, MYO18A, MYO1E, MYO5A, MYO7B, MYPN, MYRF, NAAA, NAV2, NCEH1, NDRG1, NEB, Nebl, NEDD4L, Nes, NEUROD4, NFIL3, NGFR, NKD1, NOL3, NOTUM, NOV, NPAS2, NPC1, NPR1, NPR3, NRCAM, NRK, NSUN3, NTM, NTNG2, NUMBL, OAF, OLFML1, OLFML3, Olfr920, OLIG1, OPALIN, OPLAH, Orm1 (includes others), OS9, OSR2, OTULIN, PADI2, PALMD, PANX1, PAQR6, PARP3, PCDH19, PCDH8, PCGF5, PDGFA, PDP1, PENK, PEX5L, PFKFB4, PFKP, PIEZO2, PIGZ, PIR, PKP4, PLA2G16, PLA2G2D, PLAC8, PLAUR, PLEC, PLEKHA1, PLEKHA2, PLEKHB1, PLEKHM3, PLIN2, PLOD3, PLP1, PLXNB3, PPFIBP2, PPL, PPP1R15A, PRDM16, PRICKLE1, PRKCB, PRKG1, PROS1, PRR5L, PRR7, PRRX2, PRSS35, PTPLA, PTPN21, Pvr, QPCT, RAI2, RASGEF1C, RASSF4, RBM24, RDH5, RFX3, RGCC, RGMA, RGS4, RHOU, RTKN, RUNX2, RXFP3, S1PR1, SAMD4A, SATB1, SBNO2, SCN7A, SCRN1, SCUBE1, SDC1, SEMA3B, SEMA3E, SEMA4F, SERPINA3, Serpina3h, SERPINB6, SFXN3, SFXN5, SGK1, SGK223, SH3BP2, SH3D19, SHPK, SIK3, SIRPA, SLC10A4, SLC13A3, SLC16A10, SLC16A2, SLC22A23, SLC24A2, SLC29A4, SLC35F6, SLC36A1, SLC37A1, SLC4A4, SLC4A8, SLC6A19, SLC6A6, SLC6A7, SLC9A9, SLITRK6, SMURF1, SNX10, SNX16, SNX24, SOCS2, SORCS2, SPATA13, SPHK1, SRGAP1, SRPK2, SSPN, ST18, ST6GAL1, STAC2, STARD13, STC2, STEAP3, STON2, STXBP3, Stxbp3b, SV2C, SVEP1, SYNE2, SYT6, SYTL2, TAGLN, TECTA, TENM3, TG, TGFA, TGFBI, TGM1, TGM2, TINAGL1, TIPARP, TLE3, TMEFF2, TMEM158, TMEM88B, TMOD1, TNC, TNFRSF12A, TNFRSF19, TNIK, TNMD, TNNI1, TNS3, TPBG, TPPP3, TRIB1, TRIM13, TSPAN11, TSPAN15, TSPAN17, TSPAN2, TSPO, TST, TTC12, TTC39C, TTC9, TTYH1, TUBB2A, TUBB2B, TUBB3, TXNDC16, UCN2, UGDH, UGP2, UGT1A9 (includes others), UNC13B, UNC13C, UTRN, VASH1, VSTM2B, VWA5A, WBSCR17, WIPF1, WISP1, WNT7B, XAF1, XIRP1, XKR4, YIPF1, Zfp185, ZNF469

## Supplementary Table 2: Cell Cycle / Apoptosis

| Gene | Encoded protein | Fold change | Cell Cycle |
|---|---|---|---|
| AURKA | aurora kinase A | -2.712 | STOP |
| AURKB | aurora kinase B | -4.146 | STOP |
| BORA | bora. aurora kinase A activator | -2.319 | STOP |
| BRINP1 | bone morphogenetic protein/retinoic acid inducible neural-specific 1 | -2.090 | GO |
| BTC | betacellulin | 5.946 | GO |
| BUB1 | BUB1 mitotic checkpoint serine/threonine kinase | -5.736 | STOP |
| BUB1B | BUB1 mitotic checkpoint serine/threonine kinase B | -3.464 | STOP |
| CASC5 | cancer susceptibility candidate 5 | -4.295 | STOP |
| CCNA2 | cyclin A2 | -4.652 | STOP |
| CCNB1 | cyclin B1 | -5.752 | STOP |
| | | -5.820 | |
| | | -5.857 | |
| CCNB2 | cyclin B2 | -3.392 | STOP |
| CCND1 | cyclin D1 | -2.476 | STOP |
| CCND3 | cyclin D3 | -1.539 | STOP |
| CCNE1 | cyclin E1 | -1.777 | STOP |
| CCNE2 | cyclin E2 | -2.847 | STOP |
| CCNF | cyclin F | -3.211 | STOP |
| CDC6 | cell division cycle 6 | -1.936 | STOP |
| CDC20 | cell division cycle 20 | -3.113 | STOP |
| CDC25B | cell division cycle 25B | -1.636 | STOP |
| CDC25C | cell division cycle 25C | -2.414 | STOP |
| CDC45 | cell division cycle 45 | -1.769 | STOP |
| CDCA2 | cell division cycle associated 2 | -3.461 | STOP |
| CDCA3 | cell division cycle associated 3 | -3.003 | STOP |
| CDCA5 | cell division cycle associated 5 | -3.053 | STOP |
| CDCA7L | cell division cycle associated 7-like | -4.123 | STOP |
| CDCA8 | cell division cycle associated 8 | -3.467 | STOP |
| CDK1 | cyclin-dependent kinase 1 | -3.227 | STOP |
| CDK5R1 | cyclin-dependent kinase 5, regulatory subunit 1 (p35) | 1.597 | - |
| CENPA | centromere protein A | -1.895 | STOP |
| CENPE | centromere protein E, 312kDa | -4.140 | STOP |
| CENPF | centromere protein F, 350/400kDa | -3.927 | STOP |
| CENPI | centromere protein I | -2.899 | STOP |
| CENPK | centromere protein K | -2.813 | STOP |
| CENPL | centromere protein L | -1.864 | STOP |
| CENPM | centromere protein M | -3.407 | STOP |
| CENPN | centromere protein N | -2.465 | STOP |
| CENPU | centromere protein U | -1.624 | STOP |
| CEP55 | centrosomal protein 55kDa | -3.625 | STOP |
| CHAF1B | chromatin assembly factor 1, subunit B (p60) | -2.440 | STOP |
| CHEK1 | checkpoint kinase 1 | -3.096 | GO |
| CHEK2 | checkpoint kinase 2 | -1.997 | GO |

| | | | |
|---|---|---|---|
| CKAP2 | cytoskeleton associated protein 2 | -3.765 | STOP |
| CKAP2L | cytoskeleton associated protein 2-like | -3.219 | STOP |
| CKS2 | CDC28 protein kinase regulatory subunit 2 | -2.948 | STOP |
| | | -2.878 | |
| | | -2.887 | |
| DBF4 | DBF4 zinc finger | -2.249 | STOP |
| DNA2 | DNA replication helicase/nuclease 2 | -2.242 | STOP |
| DTL | denticleless E3 ubiquitin protein ligase homolog (Drosophila) | -2.940 | STOP |
| E2F1 | E2F transcription factor 1 | -1.977 | STOP |
| E2F2 | E2F transcription factor 2 | -1.693 | STOP |
| E2F3 | E2F transcription factor 3 | -1.555 | STOP |
| EAPP | E2F-associated phosphoprotein | 1.532 | GO |
| ECT2 | epithelial cell transforming 2 | -4.764 | STOP |
| EME1 | essential meiotic structure-specific endonuclease 1 | -2.032 | STOP |
| FBXW7 | F-box and WD repeat domain containing 7, E3 ubiquitin protein ligase | -1.517 | - |
| FOXM1 | forkhead box M1 | -3.412 | STOP |
| GADD45A | growth arrest and DNA-damage-inducible, alpha | 2.711 | STOP |
| GADD45B | growth arrest and DNA-damage-inducible, beta | 2.584 | STOP |
| GADD45G | growth arrest and DNA-damage-inducible, gamma | 2.145 | STOP |
| GAS1 | growth arrest-specific 1 | 1.930 | STOP |
| GTSE1 | G-2 and S-phase expressed 1 | -2.995 | STOP |
| Hmga2 | high mobility group AT-hook 2 | -1.905 | STOP |
| INCENP | inner centromere protein antigens 135/155kDa | -2.416 | STOP |
| IQGAP3 | IQ motif containing GTPase activating protein 3 | -4.269 | STOP |
| JDP2 | Jun dimerization protein 2 | 1.688 | GO |
| JUN | jun proto-oncogene | 1.646 | GO |
| KIF2C | kinesin family member 2C | -3.648 | STOP |
| KIF4A | kinesin family member 4A | -4.520 | STOP |
| KIF11 | kinesin family member 11 | -4.722 | STOP |
| KIF14 | kinesin family member 14 | -2.833 | STOP |
| KIF15 | kinesin family member 15 | -3.948 | STOP |
| KIF18A | kinesin family member 18A | -1.895 | STOP |
| KIF18B | kinesin family member 18B | -5.056 | STOP |
| KIF20A | kinesin family member 20A | -4.189 | STOP |
| KIF20B | kinesin family member 20B | -2.338 | STOP |
| KIF22 | kinesin family member 22 | -3.063 | STOP |
| KIF23 | kinesin family member 23 | -3.710 | STOP |
| KIF24 | kinesin family member 24 | -2.141 | STOP |
| KNTC1 | kinetochore associated 1 | -2.976 | STOP |
| MASTL | microtubule associated serine/threonine kinase-like | -2.849 | GO |
| MCM2 | minichromosome maintenance complex component 2 | -2.127 | STOP |
| MCM3 | minichromosome maintenance complex component 3 | -2.604 | STOP |
| MCM4 | minichromosome maintenance complex component 4 | -1.721 | STOP |
| MCM5 | minichromosome maintenance complex component 5 | -2.409 | STOP |
| MCM7 | minichromosome maintenance complex component 7 | -1.807 | STOP |
| MCM8 | minichromosome maintenance complex component 8 | -1.729 | STOP |

| | | | |
|---|---|---|---|
| MCM10 | minichromosome maintenance complex component 10 | -2.304 | STOP |
| MELK | maternal embryonic leucine zipper kinase | -3.429 | STOP |
| MKI67 | marker of proliferation Ki-67 | -5.081 | STOP |
| NCAPD2 | non-SMC condensin I complex, subunit D2 | -2.507 | STOP |
| NCAPD3 | non-SMC condensin II complex, subunit D3 | -1.662 | STOP |
| NCAPG | non-SMC condensin I complex, subunit G | -4.432 | STOP |
| NCAPG2 | non-SMC condensin II complex, subunit G2 | -2.695 | STOP |
| NCAPH | non-SMC condensin I complex, subunit H | -3.621 | STOP |
| NDC80 | NDC80 kinetochore complex component | -2.428 | STOP |
| NEK2 | NIMA-related kinase 2 | -3.020 | STOP |
| Nes | nestin | 2.413 | GO |
| NSL1 | NSL1, MIS12 kinetochore complex component | -1.689 | STOP |
| NUF2 | NUF2, NDC80 kinetochore complex component | -4.739 | STOP |
| PARP3 | poly (ADP-ribose) polymerase family, member 3 | 2.803 | STOP |
| PBK | PDZ binding kinase | -3.672 | STOP |
| PDE3A | phosphodiesterase 3A, cGMP-inhibited | -1.781 | STOP |
| PDE3B | phosphodiesterase 3B, cGMP-inhibited | -1.860 | STOP |
| PLK1 | polo-like kinase 1 | -4.320 | STOP |
| PLK2 | polo-like kinase 2 | -2.042 | STOP |
| PLK4 | polo-like kinase 4 | -1.854 | STOP |
| POLA1 | polymerase (DNA directed), alpha 1, catalytic subunit | -1.966 | STOP |
| POLA2 | polymerase (DNA directed), alpha 2, accessory subunit | -1.593 | STOP |
| POLD1 | polymerase (DNA directed), delta 1, catalytic subunit | -1.506 | STOP |
| POLE | polymerase (DNA directed), epsilon, catalytic subunit | -3.304 | STOP |
| POLE2 | polymerase (DNA directed), epsilon 2, accessory subunit | -1.991 | STOP |
| POLH | polymerase (DNA directed), eta | -1.542 | STOP |
| PRC1 | protein regulator of cytokinesis 1 | -3.160 | STOP |
| PRIM1 | primase, DNA, polypeptide 1 (49kDa) | -1.983 | STOP |
| PSRC1 | proline/serine-rich coiled-coil 1 | -2.235 | STOP |
| RAD51 | RAD51 recombinase | -2.508 | STOP |
| RAD51AP1 | RAD51 associated protein 1 | -2.093 | STOP |
| RAD51B | RAD51 paralog B | -1.531 | STOP |
| RAD51C | RAD51 paralog C | -1.572 | STOP |
| RAD54B | RAD54 homolog B (S, cerevisiae) | -2.299 | STOP |
| RAD54L | RAD54-like (S, cerevisiae) | -2.114 | STOP |
| RFC4 | replication factor C (activator 1) 4, 37kDa | -2.030 | STOP |
| RFWD3 | ring finger and WD repeat domain 3 | -1.520 | - |
| | | -1.534 | |
| RGCC | regulator of cell cycle | 1.604 | - |
| RND3 | Rho family GTPase 3 | -1.514 | STOP |
| SATB1 | SATB homeobox 1 | 1.980 | GO |
| SHCBP1 | SHC SH2-domain binding protein 1 | -3.297 | STOP |
| SIK3 | SIK family kinase 3 | 1.780 | - |
| SKA1 | spindle and kinetochore associated complex subunit 1 | -1.532 | STOP |
| SKA2 | spindle and kinetochore associated complex subunit 2 | -1.582 | STOP |
| SKA3 | spindle and kinetochore associated complex subunit 3 | -3.490 | STOP |
| SKP2 | S-phase kinase-associated protein 2, E3 ubiquitin protein ligase | -1.845 | STOP |

| | | | |
|---|---|---|---|
| SLBP | stem-loop binding protein | -1.567 | STOP |
| | | -1.677 | |
| SMC2 | structural maintenance of chromosomes 2 | -2.408 | STOP |
| SMC4 | structural maintenance of chromosomes 4 | -1.723 | STOP |
| SPAG5 | sperm associated antigen 5 | -3.782 | STOP |
| SPC25 | SPC25, NDC80 kinetochore complex component | -4.148 | STOP |
| STIL | SCL/TAL1 interrupting locus | -4.020 | STOP |
| TACC3 | transforming, acidic coiled-coil containing protein 3 | -2.153 | STOP |
| TICRR | TOPBP1-interacting checkpoint and replication regulator | -3.857 | STOP |
| TONSL | tonsoku-like, DNA repair protein | -1.617 | STOP |
| TOP2A | topoisomerase (DNA) II alpha 170kDa | -4.814 | STOP |
| TOPBP1 | topoisomerase (DNA) II binding protein 1 | -1.626 | STOP |
| TPX2 | TPX2, microtubule-associated | -4.151 | STOP |
| TROAP | trophinin associated protein | -3.122 | STOP |
| TTK | TTK protein kinase | -3.305 | STOP |
| TYMS | thymidylate synthetase | -1.971 | STOP |
| UBE2C | ubiquitin-conjugating enzyme E2C | -3.311 | STOP |
| UBE2L6 | ubiquitin-conjugating enzyme E2L 6 | -1.675 | STOP |
| UBE2T | ubiquitin-conjugating enzyme E2T (putative) | -1.689 | STOP |
| UHRF1 | ubiquitin-like with PHD and ring finger domains 1 | -3.159 | STOP |
| USP1 | ubiquitin specific peptidase 1 | -2.027 | STOP |
| WEE1 | WEE1 G2 checkpoint kinase | -1.908 | GO |
| **Apoptosis** | | | |
| BCL3 | B-cell CLL/lymphoma 3 | 2.320 | SURVIVAL |
| BMF | Bcl2 modifying factor | 1.616 | DEATH |
| CARD6 | caspase recruitment domain family, member 6 | -1.593 | DEATH |
| CHEK1 | checkpoint kinase 1 | -3.096 | SURVIVAL |
| CHEK2 | checkpoint kinase 2 | -1.997 | SURVIVAL |
| E2F7 | E2F transcription factor 7 | -2.504 | SURVIVAL |
| E2F8 | E2F transcription factor 8 | -3.524 | SURVIVAL |
| EGLN3 | egl-9 family hypoxia-inducible factor 3 | -1.624 | SURVIVAL |
| G2E3 | G2/M-phase specific E3 ubiquitin protein ligase | -1.648 | DEATH |
| NOL3 | nucleolar protein 3 (apoptosis repressor with CARD domain) | 1.711 | SURVIVAL |
| PDCD4 | programmed cell death 4 (neoplastic transformation inhibitor) | -1.524 | SURVIVAL |
| PIDD1 | p53-induced death domain protein 1 | -1.935 | SURVIVAL |
